# Equity in dental care among Canadian households

**DOI:** 10.1186/1475-9276-10-14

**Published:** 2011-04-16

**Authors:** Carlos Quiñonez, Paul Grootendorst

**Affiliations:** 1 Discipline of Dental Public Health, Faculty of Dentistry, University of Toronto, Toronto, Canada; 2 Division of Clinical, Social and Administrative Pharmacy, Leslie Dan Faculty of Pharmacy, University of Toronto, Canada

## Abstract

**Background:**

Changes in third party financing, whether public or private, are linked to a household's ability to access dental care. By removing costs at point of purchase, changes in financing influence the need to reach into one's pocket, thus facilitating or limiting access. This study asks: How have historical changes in dental care financing influenced household out-of-pocket expenditures for dental care in Canada?

**Methods:**

This is a mixed methods study, comprised of an historical review of Canada's dental care market and an econometric analysis of household out-of-pocket expenditures for dental care.

**Results:**

We demonstrate that changes in financing have important implications for out-of-pocket expenditures: with more financing come drops in the amount a household has to spend, and with less financing come increases. Low- and middle-income households appear to be most sensitive to changes in financing.

**Conclusions:**

Alleviating the price barrier to care is a fundamental part of improving equity in dental care in Canada. How people have historically spent money on dental care highlights important gaps in Canadian dental care policy.

## Background

Equity in dental care use has recently gained more prominence as a health policy issue in Canada and in other OECD nations [1-5]. Among these countries, Canada ranks second last in the public financing of dental care [6]. As opposed to its national system of public health insurance, dental care in Canada is almost wholly privately financed, with approximately 60% of dental care paid through employment-based insurance, and 35% through out-of-pocket expenditures [7,8]. Of the approximately 5% of publicly financed care that remains, most has focused on socially marginalised groups (e.g. low income children and adults), and is supported by different levels of government depending on the group insured [9].

In this context, Canadian governments are now being asked to respond to historical and emerging issues in access to dental care [9]. Significant inequalities in oral health and access to care are long-standing and well documented [8,10]. It is known that socially marginalised groups experience disproportionate levels of oral disease, illness, and disability, and are the least likely to visit a dentist or have dental insurance. New populations and access problems are also appearing, such as 'the working poor', who do not qualify for public insurance, yet do not have jobs that offer employment-based insurance, again the country's dominant form of financing care [11,12]. Further, it is anecdotally reported that in light of the recent global economic downturn, middle-income families are now contacting local public health agencies in an effort to access publicly financed care.

Overall, Canada's dental care system has been defined as inequitable on a variety of fronts. For example, as Leake [13] has stressed, Canada's dental care system is a clear example of 'the inverse care law,' where the people that need the most care receive the least. Allin [14], using the horizontal index approach (which tests the horizontal version of the equity principle, requiring that people in equal need of care are treated equally, irrespective of characteristics such as income, place of residence, race, etc.), has demonstrated that Canada's dental care system is 'pro-rich,' confirming that in all of its ten provinces, the probability of visiting a dentist is much higher for those with the least need. Similar work by van Doorslaer and Masseria [15] shows that Canada ranks among the poorest performers among OECD nations in this regard. Allin [14] also notes that the main contributors to inequity in dental care are income and dental insurance coverage. Lastly, the nature of dental care financing in Canada has also been heavily criticized relative to its shortcomings in the face the policy push towards health care equity [13]. As mentioned, dental care is predominantly financed through employer-employee arrangements. These 'non-wage benefits' are tax subsidized by governments, meaning that these benefits do not attract income tax. This results in a situation where those with the least amount of need and economic barriers to care pay for care with pre-tax dollars, while those with the most need and the greatest economic barriers (i.e. low-income and no employer provided dental insurance) pay with after tax dollars. In short, the poor to some extent subsidize the rich [13].

This paper presents another way of exploring the issue of equity in dental care through the use of Engel curves, a way of demonstrating how the quantity demanded of a good or service changes as income level changes. In one sense, Engel curves are an indirect assessment of equity in financing, and how this relates to access to dental care, specifically in terms of how affordability influences the ability of people to access care. This analysis is predicated on one general assumption, that household spending is related to access through the issue of affordability. By removing costs at point of purchase, changes in third party financing, whether public or private, influence the need to reach into one's pocket, thus facilitating or limiting access to care.

Yet what is the connection between third party financing, out-of-pocket expenditures, and access to dental care? While it may appear reasonable to assume that policy changes in third party financing impact out-of-pocket expenditures, this does necessarily mean impacts on access. In fact, Leake and Birch [10] note that while removing 'the price barrier to care' is a necessary step towards improving access to dental care, it is not sufficient, as access also depends on such things as provider availability, consumer behaviour, and third party fee arrangements. Nevertheless, the connection is a close one. For example, in Canada, it is known that cost consistently ranks as the second most prevalent reason for not visiting a dentist, and first for those with no dental insurance [8]. It is also known that the financing of dental care through public or private insurance represents a strong determinant of dental care utilization [8,16]. It makes sense to argue that household out-of-pocket expenditures for dental care represent a marker for insurance, meaning that the more insurance a household has, the less of a share of its budget it needs to commit to dental care. One can further argue that in an insurance rich market, such as Canada, out-of-pocket expenditures can represent a reasonable proxy of access, in that the more a household has to spend, the more difficult it may actually be to access care, all things being equal.

Recent data from Locker et al. [17] suggest that the presence of third party financing substantially reduces, yet does not entirely eliminate financial barriers to dental care (as measured by a series of questions, "In the past three years...has the cost of dental care been a financial burden to you?...have you delayed or avoided going to a dentist because of the cost?...have you been unable to have all of the treatment recommended by your dentist because of the cost?"). For the lowest income group and those who paid out-of-pocket, the most common concern expressed was the financial burden imposed by the costs of dental care. For the highest income group and those with private insurance coverage, not being able to have all the treatment recommended by a dentist was the most frequently expressed concern. At minimum, what this tells us is that third party financing influences how deeply individuals must reach into their pockets to pay for dental care, thus promoting or limiting access to care generally and specifically.

These ideas are corroborated in studies of access to health and dental care in other international contexts. For example, Berk and Schur [18] demonstrated that in the United States, it is the uninsured that report a financial inability to access health and dental care, more so than those with public (Medicaid) or private insurance. Long [19] also showed that in one state, after the restoration of dental and other health benefits through Medicaid, there came a drop in the share of adults reporting high out-of-pocket costs and problems paying medical and dental bills, a decrease in those that reported not accessing dental care because of cost, and an increase in the number of low-income adults with a dental care visit. In Thailand, which recently implemented universal financing for dental care, Somkostra and Detsomboonrat [20] showed that after implementation, there was an increased likelihood among the poor for accessing and utilizing dental services at public and private facilities. Conversely, Falkingham [21] demonstrated that in Tajikistan, which initiated reform to secure financing for health services through non-budgetary sources such as voluntary insurance and patient cost-sharing, such activity has deterred people from seeking medical assistance and from receiving the most appropriate treatment once medical advice has been sought. Even amongst the third and fourth income quintiles of Tajikistan households, assets are sold and debt incurred in order to meet the costs of health and dental care. Similarly, in Canada, Muirhead et al. [22] have demonstrated that working poor families make important budgetary trade-offs, sometimes involving food, in order to fulfil their dental care needs.

Ultimately, in order to inform policy discussions around the issue of equity in dental care, this paper asks: How have historical changes in dental care financing influenced household out-of-pocket expenditures for dental care in Canada? At minimum, Engel curves allow for an assessment of the distributional impacts of changes in dental care policy on out-of-pocket expenditures, or how households of different levels of affluence benefit from various policies and programs.

## Methods

This is a mixed methods study, comprised of an historical review of Canada's dental care market and an econometric analysis of household out-of-pocket expenditures for dental care. All components received approval from the University of Toronto's ethics review board.

### Historical review

Changes to the structure of Canada's dental care market were established through: 1. A review of the complete series of the Journal of the Canadian Dental Association (1935-2008), the Canadian Society for Public Health Dentistry Journal (1980-1985), and the Canadian Journal of Community Dentistry (1986-2000); 2. A review of electronically available governmental and non-governmental documentation (e.g. ministry and departmental annual reports, scholarly publications).

To describe changes in dental care financing, health expenditure data from the Canadian Institute for Health Information and historical population estimates from Statistics Canada were used to plot per capita dental care expenditures from 1975-2005 (constant dollars) nationally. To extend this timeline into the 1960s, estimates within the dental literature were used [23].

### Engel curves

To observe the impacts of changes in dental care financing on out-of-pocket expenditures for dental care, Engel curves were created. Data on out-of-pocket contributions for dental care from the Survey of Family Expenditures and the Survey of Household Spending were used. Both surveys record detailed annual spending patterns for a nationally representative sample of residential households in Canada. Data were used for years 1969, 1982, 1992, 1998, and 2003.

Semi-parametric regression models of household out-of-pocket expenditures for dental care were estimated as a function of household budget, holding constant other household characteristics (age, sex, and marital status of the household head and (log) household size). All expenditures were adjusted using the national all-item consumer price index (2002 = 100). Models were estimated of the form:(1)

where *dxshare*[i] = dental care share of household i's budget; *budget*[i] = the log of household i's budget, and f(*budget*[i]) is an unknown function to be estimated non-parametrically; **beta' **is a set of unknown parameters to be estimated; ***x***[i] = a set of characteristics of household i including the age, sex, and marital status of the household head and (log) household size; and *u*[i] = combined effect of all other factors besides *budget*[i] and ***x***[i] on *dxshare*[i]. Here, the impact of *budget*[i] on *dxshare*[i] is not restricted whereas the impact of ***x***[i] on *dxshare*[i] is restricted as per the conventional linear regression model. The model was estimated using the procedure recommended by Robinson [24]. One first estimates **beta' **by a) removing the influence of *budget*[i] from *dxshare*[i] and ***x***[i] non-parametrically, resulting in transformed variables that we will call dxshare*[i] and x*[i]; and then b) estimating the linear regression of dxshare*[i] and x*[i]. Second, one then subtracts **betahat'*x***[i] from both sides of equation (1), where **betahat' **are the estimated values of **beta'**. Hence the LHS of equation (1) becomes *dxshare*[i]- **betahat'*x***[i]; and *budget*[i] is the sole explanatory variable. One then estimates the relationship between these two variables using a local smoothing technique to arrive at the estimate of f(*budget*[i]).

Models were estimated separately for each year, allowing us to compare the dental care budget shares of comparable households at different points in time. Hence we could determine how changes in third party coverage have affected the dental care budget shares of both affluent households (i.e. those with large household budgets) and less affluent households. Moreover, we could compare, for a given year, how dental care budget shares varied across affluent and less affluent households.

## Results

### Changes in public financing

**Figure 1:**
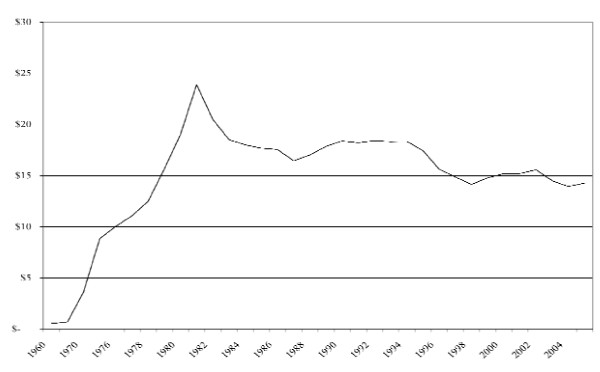
**Public per capita dental care expenditures, Canada, 1960-2005 (constant dollars)**. Source: Historical Statistics of Canada, Social Science Federation of Canada; National Health Expenditure Database, Canadian Institute for Health Information; National population estimates, Statistics Canada; Historical inflation rates, Bank of Canada.

**Figure 2:**
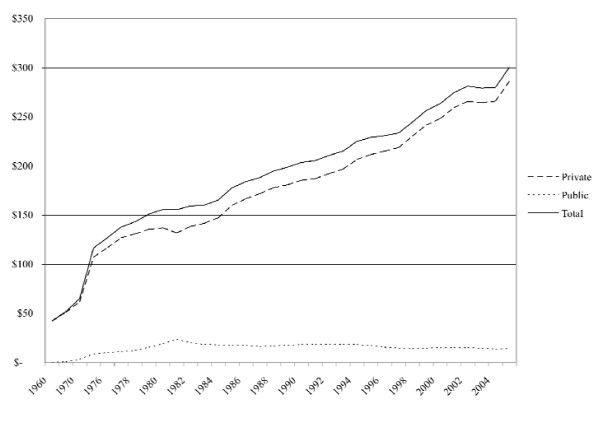
**Total, private, and public per capita dental care expenditures, Canada, 1960-2005 (constant dollars)**. Source: Historical Statistics of Canada, Social Science Federation of Canada; National Health Expenditure Database, Canadian Institute for Health Information; National population estimates, Statistics Canada; Historical inflation rates, Bank of Canada.

Governmental investments in dental care effectively began after WWII as part of the rise of the Canadian welfare state. In 1948, federal 'health grants' were made available to provincial jurisdictions, which included investments for public dental infrastructure [25]. By 1967, Canada had nationalised hospital and physician services, giving rise to the country's national system of universal health insurance, yet this excluded dental services [26]. Dental services were instead publicly insured in a targeted fashion, specifically for children and those receiving social transfers (e.g. welfare recipients) [27]. In this regard, federal contributions facilitated provincial investments in children's and social assistance programs, delivered directly and/or indirectly. Figure 1 demonstrates that these investments grew rapidly throughout the 1960s and 1970s. Comparatively though, Figure 2 demonstrates that Canada's investments in dental care were largely based on private financing.

At the height of public financing in the early 1980s, the public share for dental care stood at approximately 20%, but has since observed a general decline (Figure 1). This decline was defined by an economic recession in the early 1980s, followed by another recession in the mid 1990s, both resulting in major reductions to government financing [9]. Direct delivery programs were cut, eligibility criteria were stiffened, and the basket of available services was shrunk.

Throughout this decline, there have been some modest rebounds, almost exclusively in relation to children's programs, with more funds made available to increase fees and/or expand eligibility [9]. For example, the federal National Child Benefit was introduced in the mid 1990s, which resulted in provincial investments for children of low-income families. Other jurisdictions have also introduced funding for seniors and refugees.

### Changes in private financing

The growth of private financing was also linked to the rise of the welfare state. Prior to 1967, there was little private dental insurance in Canada, with most private financing composed of individual out-of-pocket expenditures [23]. Yet with the exclusion of dental services from the nation's system of health insurance, the private financing of dental care took shape. With the growth of unionization, dental benefit plans became a major part of employer-employee contracts [27-29]. This was facilitated by provincial subsidies in the form of taxation policy that excluded non-wage benefits such as dental insurance from payroll taxes [30]. For example, close to 5,000 contracts were in force in 1976, and 18,000 by 1982 [31,32]. These investments are well reflected in Figure 2 from the 1970s onwards.

The private financing of dental care stuttered with the economic recession of the 1980s, yet remained in steady incline. While growth in wages and salaries stagnated, this did not slow supplementary labour income (non-wage benefits) [33]. From 1967 to 1989, supplementary labour income growth rates consistently outranked those of wages and salaries, almost doubling the share of total compensation from 5% to 10%. Also, the share of supplementary labour income made up by dental plans rose consistently, from 23.8% in 1967 to 30% in 1989.

**Figure 3:**
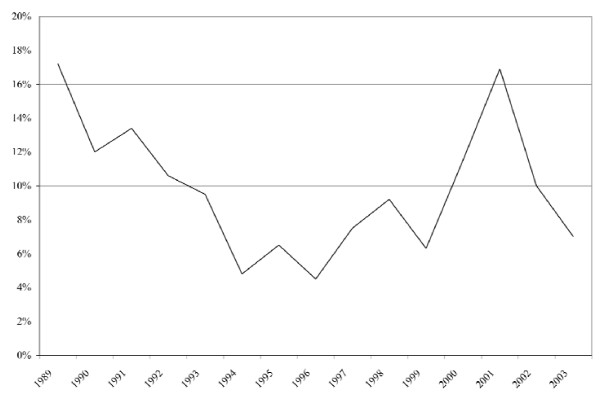
**Private health insurance expenditures, annual growth rates, Canada, 1989-2003**. Source: National Health Expenditure Database, Canadian Institute for Health Information.

Yet by the 1990s recession, employment-based insurance began to suffer. Canadian firms began to search for ways to cost-contain, and benefit plans were changed to limit annual maximums and services, and/or by introducing or expanding deductibles, co-insurance or co-payments [34,35]. Preventive services were also 'bundled', so rather than a fee charged for each procedure, a single relative value was applied to various combinations of services, reducing the overall fee [35]. 'Flex benefit plans' were also introduced, which no longer defined maximums for a set of benefits (e.g. dental, vision, supplementary medical), but instead allowed employees to choose the type and amount of coverage desired from a variety of services [36]. This allowed employers to provide more and different services, while limiting increases in expenditures. The volatility in insurance markets during this period is observable in Figure 3.

Canadian firms also began to change the nature of employment in response to economic constraints and growing global competition [37]. Unionisation slowed, which had grown from approximately 2 million persons in 1967 to 3.8 million in 1990, yet totalled 3.5 million by 1997 [28]. Firms reduced their wage offers to new employees, and offered temporary jobs to a growing proportion of new employees. The fraction of new employees in temporary jobs rose from 11% in 1989 to 21% in 2004. Importantly, part-time and temporary full-time workers generally receive fewer benefits than regular full-time workers [38]. Finally, changes have been differentially distributed in the employee population, meaning that low and middle-income employees have observed the greatest impacts to their workplace structures and non-wage offers [28,37,38].

### Impacts on household out-of-pocket expenditures for dental care

In the earliest year, 1969, there was little private or public coverage of dental care services, so that most expenditures were made out-of-pocket. During this time, the share of the household budget allocated to dental care was small (less than 1 percent) but nevertheless much higher for more affluent households. Dental care therefore acted like a luxury good. Since 1969 there has been an expansion of the range and quality of dental care services offered, a commensurate increase in their prices, and increase in third party coverage. Between 1969 and 1982, there was a drop in the dental care budgetary share of less affluent households - those with budgets less than $15,000; this is likely due to the expansion of public dental subsidies for the poor observed in Figure 1. Since 1982, there has been a retrenchment in public subsidies for the poor, and this would increase their budget share proportionally if their use of dental care remained constant. Yet we know that the dental care services use of less affluent households is particularly price sensitive so that the reductions in public subsidies since 1982 has reduced the use of services among such households and thus also mitigated the post-1982 growth in their budget share.

The situation for more affluent households is different. Expansion of third party coverage has resulted in a sharp drop in their budget share up until 1992. The decline in financial burden of dental care services for such households is much larger than the decline observed for less affluent households between 1969 and 1982. There have been slight increases in the budget share of affluent households since then, likely to do with the availability of generally uninsured cosmetic procedures and the limits placed on non-wage benefits.

## Discussion

**Figure 4:**
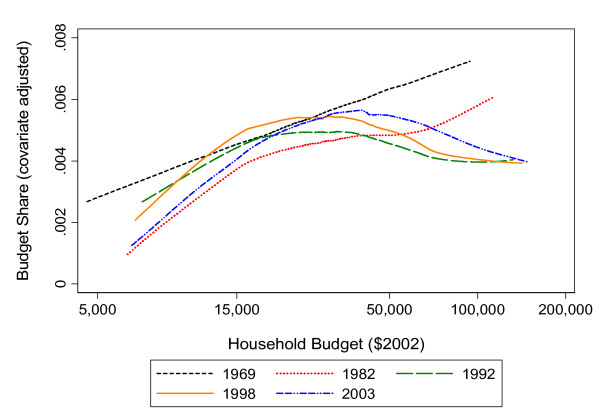
**Household budgetary shares for dental care, by household budget, Canada, 1969-2003**. Note: x axis is measured in the log scale

Changes in dental care financing are strongly linked to out-of-pocket expenditures for dental care. This is best observed in Figure 4 through the two periods comprising 1969 to 1992, where major investments in public and private financing resulted in clear and logical impacts to the household budgetary share for dental care: with more financing came drops to the household share, and with less financing came increases. The period 1992 to 1998 is less clear. It is unknown whether the modest public investments at the time had an impact, or whether drops in the household share for those of very low income is due to a decreased financial capacity during difficult economic times. The period from 1998 to 2003 is the least clear for similar reasons, meaning that a drop for all low-income households is arguably not explained by the very modest public investments made at the time, especially in an overall period of economic decline.

Yet what is most clear about the Engel curves is that the magnitude of fluctuations for low to middle income families is greatest, arguably making these families the most sensitive to changes in dental care financing. This idea is supported by recent research into the oral health and access to care disparities associated with working poverty in Canada [11,12,22]. Recent data also demonstrates that when compared to the public and privately insured, those paying for dental care out-of-pocket are more likely to report painful aching in their mouths, and more likely to report staying in bed in the previous two-weeks because of dental pain [11].

Given this, it is important to consider the central limitation of our analysis. Namely, changes to household expenditures can be explained by numerous factors: the presence of third party financing, changes in the share that an insured household must pay, changes in utilisation, in pricing, and in general economic conditions. This means that our interpretations are not considered causal, but instead are hypothesis generating. We assume that third party financing influences how deeply individuals must reach into their pockets to pay for dental care, thus facilitating or limiting access to care. Yet the obverse may also be true, for example, if providers plan care in relation to what is covered by an insurance plan, treatment may involve more extensive services at greater cost, so the relationship between household expenditures and access is definitely not clear-cut. This also depends on the price sensitivity of dental services use. If a household is very price sensitive then they will not consume any dental services that costs them money; we cannot distinguish such a household from one that has used a lot of dental services whose cost was picked up by a third party. In other words, households with low dental services expenditures can be quite different.

It is also important to consider international work in this area, yet from our review, nothing like this exists in the dental literature. Nevertheless, the use of Engel curves to explore the impacts of changing social and economic conditions on the consumption of goods such as food and pharmaceuticals is well established [39-42]. Engel curves provide useful information regarding consumption patterns across incomes, thus facilitating inferences on the distributional impacts of changing social conditions.

In terms of the policy implications of our findings, it is clear that changes in dental care financing are important for the ability of households to access dental care. Thus as a policy instrument, removing the price barrier to care is fundamental [10]. Yet specifically to Canada, how this price barrier is removed warrants attention. Leake and Birch [10] state that the net effect of Canada's method of financing dental care is a 'perversion': since employment based insurance is present for those with stable jobs and incomes, and since dental insurance is excluded from payroll taxation, those with insurance (the rich) pay for dental care using pre-tax dollars, and those with no insurance (the poor) pay with after-tax dollars. As stated in our introduction, this means that the poor in effect subsidise the rich, representing a damning view of the wealth transfer principle in the Canadian welfare state as it applies to dental care. Moreover, in the context of available public insurance, which is targeted mainly at children, and social policy that largely functions on the bases of 'deserving and undeserving poverty' [43], this magnifies the need for public subsidies among working poor families. Policy leaders should thus pay closer attention to the changing nature of employment and explore policy and legislative instruments that aim to secure or promote non-wage benefits for low income and/or temporary work arrangements. Similarly, low-income programs will need to broaden their eligibility in order to buffer changing economic conditions for families, not just children. These recommendations also apply to countries that finance dental care in a similar manner (a combination of employment-based insurance supplemented by public subsidies for the poor), in particular the United States, and to a lesser extent countries such as Belgium, Finland, Sweden and the United Kingdom, and for those without substantial amounts of employment-based insurance, but with tax subsidies aimed at promoting the direct purchase of private dental insurance, such as Australia, the Netherlands, and France [44,45]. That said, another way to remove the price barrier to care is through universal coverage, which in most countries exists in a targeted approach, such as school based services in Chile and Brazil, or dedicated direct delivery systems such as in the United Kingdom, Sweden, and Norway [46].

## Conclusions

This study has presented a mixed methods approach to assessing the potential impacts of social, economic, and/or policy changes on household expenditures for dental care. There may be much opportunity for this approach in an international and comparative context. From the local perspective, this approach ultimately provides decision-makers with a readily interpretable visual representation of how historical changes in financing can impact expenditures for dental care, thus influencing equity in dental care in general.

## Competing interests

The authors declare that they have no competing interests.

## Authors' contributions

CQ wrote the manuscript, conducted the historical review, and interpreted the results. PG acquired the data for statistical analysis, guided this analysis, and interpreted the results. Both authors have read and approved the final manuscript.
